# Impact of Nurse-Performed Point-of-Care Ultrasound (PoCUS) in Adult Intensive Care: A Systematic Review

**DOI:** 10.3390/healthcare14101286

**Published:** 2026-05-09

**Authors:** Marco Abagnale, Chiara Palazzo, Nicolò Zampetti, Melania De Filippo, Rita Citarella, Fabio Gennaro Abagnale, Luciano Cecere, Francesco Limonti, Francesco Gravante

**Affiliations:** 1 Unit of Anesthesiology and Intensive Care, Department of Critical Care, M. Scarlato Hospital, Local Health Authority of Salerno, 84018 Scafati, Italy; abagnale.marco@gmail.com (M.A.); fabiogennaroabagnale@libero.it (F.G.A.); 2 Department of Oncology, Hematology and Cellular Therapies, Santobono Pausilipon Hospital, 80123 Naples, Italy; 3 Department of Health Care, Local Health Authority of Benevento, San Giorgio del Sannio, 82018 Benevento, Italy; zampettin@hotmail.it; 4 Colorectal Surgical Oncology, Department of Abdominal Oncology, Istituto Nazionale Tumori–IRCCS “Fondazione G.Pascale”, 80131 Naples, Italy; defilippo.melania@virgilio.it; 5 Department of Surgery and Anaesthesia, Umberto I Hospital, Local Health Authority of Salerno, 84014 Nocera Inferiore, Italy; rita.citarella.91@gmail.com; 6 Department of Anesthesia and Resuscitation, A.O.R.N. Antonio Cardarelli, 80131 Naples, Italy; luciano.cecere@aocardarelli.it; 7 Department of Pharmacy, Health and Nutritional (DFSSN), University of Calabria, 87036 Rende, Italy; francesco.limonti@unical.it; 8 Intensive Care Unit, Department of Critical Care, G. Moscati Hospital, Local Health Authority of Caserta, 81100 Caserta, Italy; fra.gravante83@gmail.com

**Keywords:** point-of-care ultrasound, intensive care unit, critical care nursing, clinical decision-making, vascular access, advanced nursing practice, diagnostic accuracy, professional autonomy

## Abstract

**Highlights:**

**What are the main findings?**

- Nurse-performed POCUS in ICU supports real-time nursing clinical reasoning and leads to management changes in a clinically relevant proportion of cases.
- With structured training, diagnostic accuracy is adequate and no increase in complications has been reported.

**What are the implications of the main findings?**

- Integrating POCUS into advanced nursing practice may strengthen professional autonomy and interdisciplinary collaboration in critical care.
- Standardized training pathways and competency frameworks are essential to ensure safe and sustainable implementation.

**Abstract:**

Background/Objectives: Point-of-care ultrasound (POCUS) is increasingly used in intensive care units (ICUs) as a rapid bedside diagnostic tool supporting timely clinical assessment. The impact of nurse-performed POCUS on clinical management, procedural performance, and professional practice in adult ICUs has not yet been systematically synthesized. This review aimed to evaluate the integration of nurse-performed POCUS into nursing care and its effects on technical, decision-making, and professional outcomes. Methods: A systematic review was conducted in accordance with PRISMA 2020 and the JBI Manual for Evidence Synthesis. PubMed, Scopus, CINAHL, and Web of Science were searched without time restrictions. Original studies were included if they involved adult ICU patients and evaluated POCUS performed by nurses, reporting clinical, procedural, or professional outcomes. Methodological quality was assessed using JBI and MMAT checklists according to study design. Results: Eleven studies were included. The results were synthesized into four primary domains: (1) support for the clinical decision-making process, (2) technical performance and procedural outcomes, (3) diagnostic accuracy, and (4) professional autonomy, training, and sustainability of competencies. Nurse-performed POCUS was associated with management changes in 26–67% of assessments and improved first-attempt success in ultrasound-guided peripheral venous access. Diagnostic accuracy was acceptable when supported by structured training, with no reported increase in complications. Conclusions: Nurse-performed POCUS in adult ICUs appears safe and practice-enhancing, supporting decision-making and selected procedural outcomes. Further multi-center controlled studies are required to clarify its impact on major clinical endpoints and long-term outcomes.

## 1. Introduction

The global burden of critical illnesses has increased significantly, largely due to population ageing, the growing prevalence of chronic diseases, and the emergence of new infectious threats, as highlighted by the COVID-19 pandemic [1]. The global annual incidence of critical illness in adults is estimated to be between 30 and 45 million cases [2], driving a growing demand for intensive care services. This trend is destined to intensify as life expectancy increases and chronic patients survive longer thanks to therapeutic advances [3]. This results in increasing pressure on Intensive Care Units (ICUs), which are called upon to ensure timely diagnoses, appropriate life-saving interventions, and the management of patients with high clinical complexity, often in resource-limited settings [4].

Point-of-care ultrasound (POCUS) is defined as a focused ultrasound examination performed and interpreted directly by the clinician at the patient’s bedside, with immediate integration into the clinical decision-making process, distinguishing it from traditional consultative imaging performed in specialist departments [5,6].

POCUS has progressively established itself as an essential component of critical care medicine. Initially introduced as procedural support, particularly for the placement of venous access, it is now widely used in hospital, pediatric, and perioperative settings. Portable, non-invasive, and repeatable, it enables real-time assessment of the cardiovascular, respiratory, and vascular status of the critically ill patient. Findings suggest benefits in terms of safety, appropriateness of decision-making, and timeliness of diagnosis [7,8,9]. Furthermore, the miniaturisation of devices and reduction in costs have promoted its spread even in resource-limited settings [10,11].

PoCUS has been increasingly incorporated into the initial evaluation of acutely ill patients, particularly in those presenting with acute dyspnea, where rapid diagnostic clarification is essential [12]. The use of PoCUS has been associated with substantial reductions in key process-related outcomes [13]. Specifically, PoCUS implementation has been linked to a reduction in time to diagnosis of approximately 1 h and in time to treatment initiation of nearly 30 min compared with conventional diagnostic pathways [13,14]. While overall in-hospital length of stay does not appear to be significantly influenced, the duration of ICU stay has been shown to decrease by slightly more than one day in patients managed with a PoCUS-integrated approach, suggesting improved early risk stratification and optimisation of critical care resources [13]. In addition to temporal outcomes, PoCUS use has been associated with more than a twofold increase in the likelihood of receiving appropriate initial therapy, reflecting enhanced diagnostic accuracy and better therapeutic alignment [15]. Conversely, available results indicate no meaningful reduction in 30-day readmission rates or short-term mortality [13,16]. Collectively, these data support the role of PoCUS in improving the efficiency of care delivery and the appropriateness of management, primarily by accelerating diagnosis and treatment rather than by directly affecting survival [13].

Although historically the prerogative of physicians, the use of PoCUS has progressively extended to other healthcare professions [17]. About 55.9% of physicians report using it in clinical practice [18], with particularly wide adoption in emergency and urgent care, internal medicine, anesthesia, cardiology, and perioperative care settings [19,20,21]. At the same time, the use of PoCUS is increasing among nurses and other non-physician professionals, especially for ultrasound-guided venous access and other imaging-guided procedures [7,22,23]. Integrative reviews document a growing use by registered nurses (RNs) and nurse practitioners (NPs) for targeted assessments and support of clinical decision-making [24]. However, the extent of implementation, scope of practice, and training pathways vary across countries and healthcare systems.

In the nursing field, vascular access is the most extensively studied application, particularly in patients with difficult intravenous access (DIVA) [25]. Randomized and observational studies show that ultrasound guidance performed by adequately trained nurses increases first-attempt success and reduces complications compared with the traditional technique [11,26,27]. Further applications include hemodynamic assessment by focused echocardiography and evaluation of the inferior vena cava to estimate fluid responsiveness [28], as well as lung ultrasound for the identification of pleural effusion, pneumothorax, and pulmonary edema [29]. PoCUS also provides procedural support for thoracentesis and central venous catheter placement, helping to improve safety [30]. The expansion of these skills is part of the evolution of advanced nursing roles and of organisational strategies aimed at optimising resources and timeliness of care.

Despite preliminary results suggesting a potential value of PoCUS in intensive care [31], the literature remains heterogeneous. Previous and ongoing evidence syntheses have explored the use of point-of-care ultrasound in critical care, often focusing on specific applications such as vascular access or diagnostic accuracy, or on professional groups. For example, an ongoing systematic review registered in PROSPERO (CRD420251121198) addresses related aspects of PoCUS use in critical care settings. However, these reviews tend to focus on narrower clinical or procedural outcomes and do not comprehensively address the broader impact of nurse-performed PoCUS across clinical, diagnostic, procedural, and professional domains.

Therefore, the present review aims to provide a more comprehensive and multidimensional synthesis of the available evidence, specifically focusing on nurse-performed PoCUS in adult intensive care settings and its role in supporting clinical decision-making, technical performance, diagnostic accuracy, and professional development. The contribution of nurse-performed PoCUS as an integrated tool within the overall care process, in nursing clinical reasoning, and in supporting technical and procedural decisions in critically ill adult patients remains largely unexplored. Furthermore, it is not clearly defined whether a structured integration of PoCUS into nursing practice in the ICU can systematically influence care pathways and nursing decision-making. A recent scoping review protocol has been published on the use of POCUS by critical care nurses, aiming to map clinical applications, training pathways, and reported impacts [17]. However, as this work represents a protocol and does not include a completed synthesis or quality appraisal of the evidence, a systematic review focusing on the clinical and organisational impact of nurse-performed POCUS in adult intensive care settings is still lacking.

### Objective

This systematic review aims to critically synthesise the available literature on nurse-performed PoCUS in adult intensive care units, examining its integration into clinical and nursing practice and exploring its potential impact on clinical decision-making, technical and procedural performance, diagnostic accuracy, as well as professional and organisational aspects.

## 2. Materials and Methods

### 2.1. Design and Reporting Standards

This study was conducted as a mixed methods systematic review, following the Joanna Briggs Institute (JBI) methodology for mixed methods systematic reviews. A convergent integrated approach was adopted to synthesise quantitative, qualitative, and mixed-methods evidence into a unified set of findings [32]. The review was reported in accordance with the Preferred Reporting Items for Systematic Reviews and Meta-Analyses (PRISMA) 2020 guidelines [33] and the JBI Manual for Evidence Synthesis [34]. The review protocol was prospectively registered in the International Prospective Register of Systematic Reviews (PROSPERO) under registration number CRD420251114795 and previously published [35]. Minor deviations from the registered protocol were made during the review process. Although the original protocol was primarily oriented towards effectiveness outcomes, the final review adopted a broader mixed methods approach, incorporating qualitative and mixed-methods evidence to provide a more comprehensive understanding of the impact of nurse-performed PoCUS. This adjustment was necessary to reflect both the heterogeneity of the available evidence and the multidimensional nature of the phenomenon under investigation, in line with JBI guidance for mixed-methods systematic reviews [36].

### 2.2. Research Question and Eligibility Criteria

The research question was formulated using the PICO (Population, Intervention, Comparator, Outcome) framework as follows:

- Population: adult patients (≥18 years) admitted to ICU and critical care nurses.
- Intervention: use of PoCUS performed by nurses in the intensive care unit as a tool to support nursing assessment, care management, and technical and procedural decision-making in clinical practice.
- Outcome: Clinical and procedural outcomes, safety and diagnostic accuracy, and professional and organisational impact of the use of nurse-performed POCUS in intensive care.

Given the mixed methods nature of this review and the heterogeneity of the included study designs, a formal comparator was not consistently applicable. Therefore, the framework was pragmatically adapted to a PIO (Population, Intervention, Outcome) structure, which is commonly used in exploratory and mixed methods systematic reviews to ensure alignment between the research question, inclusion criteria, and synthesis approach.

Based on this framework, the study aimed to answer the following research question:

“*What is the reported use and impact of nurse-performed PoCUS in the intensive care unit on nursing decision-making processes, clinical assessment, and technical–procedural management in critically ill adult patients?*”

Original primary studies with quantitative, qualitative, or mixed-methods designs were eligible for inclusion. This included randomized controlled trials, observational studies (such as cohort, cross-sectional, and case series), quasi-experimental studies, diagnostic accuracy studies, and qualitative or mixed-methods research, provided they met the following criteria: (a) involvement of adult patients (≥18 years) admitted to intensive care units; (b) focus on the use of PoCUS performed by nursing staff to support nursing assessment, care management, and technical and procedural decision-making in clinical practice; (c) studies reporting clinical outcomes (diagnostic accuracy, complications, timeliness of decision-making, patient-centred outcomes) and/or outcomes related to nursing practice (professional autonomy, feasibility, acceptability); (d) studies written in English. Only studies published in English were included due to feasibility constraints and the lack of resources for translation. While this approach may have introduced a potential language bias, it was considered necessary to ensure the accuracy and consistency of data extraction and interpretation. Studies were excluded if they had one or more of the following characteristics: (a) pediatric population or patients not admitted to adult intensive care units; (b) use of PoCUS performed exclusively by physicians or by other non-nursing professionals; (c) lack of data on clinical outcomes or outcomes related to nursing practice; (d) reviews, conference abstracts, editorials, commentaries, study protocols; (e) studies not written in English. Review articles (including systematic, scoping, and narrative reviews) were excluded from the analysis, as the aim of this study was to synthesise primary empirical evidence rather than secondary syntheses. Nevertheless, existing reviews were consulted during the preliminary phase to contextualise the current state of knowledge and to inform the positioning of the present review within the broader evidence landscape.

### 2.3. Search Strategy

A systematic search was conducted in PubMed, Scopus, CINAHL, and Web of Science, with no time restrictions. The last search was performed on 15 December 2025. The search strategy combined controlled vocabulary (Medical Subject Headings, MeSH) and free-text keywords relevant to the review question. Key terms included “point-of-care ultrasound,” “critical care nursing,” “clinical decision-making,” “intensive care units,” “workflow efficiency,” “patient safety,” “nursing autonomy,” and “bedside assessment.” Boolean operators (AND, OR) were used to combine search terms.

The search strategies were iteratively developed and refined to balance sensitivity and specificity. For transparency and reproducibility, the complete search strategy used for PUBMED is reported below: (“Intensive Care Units”[Mesh] OR “Critical Care”[Mesh] OR ICU[tiab] OR “intensive care”[tiab] OR “critical care”[tiab]) AND (“Nurses”[Mesh] OR “Nursing”[Mesh] OR nurse*[tiab] OR “critical care nurse*”[tiab] OR “intensive care nurse*”[tiab]) AND (“Ultrasonography”[Mesh] OR “Point-of-Care Systems”[Mesh] OR ultrasound[tiab] OR ultrasonograph*[tiab] OR “point-of-care ultrasound”[tiab] OR POCUS[tiab] OR “bedside ultrasound”[tiab]).

Full search strategies for all databases are provided in the Supplementary Materials (Table S1). In addition, the reference lists of all included studies were manually screened to identify further relevant articles; no additional eligible studies were identified.

### 2.4. Study Selection

The articles underwent systematic screening using the online software Rayyan (https://www.rayyan.ai, Rayyan Systems Inc., Cambridge, MA, USA, accessed on 1 December, 2025), a free web-based application designed to facilitate and accelerate the initial screening of titles and abstracts through a semi-automated process while maintaining high usability. Two reviewers (L.C. and N.Z.) independently screened all records by title and abstract using the blinded mode, which ensured that each reviewer was unaware of the other’s decisions during the selection process. The blinded mode was deactivated only after the completion of the initial screening phase to allow comparison of decisions. Any disagreements between the reviewers were resolved through discussion and consensus; when consensus could not be reached, a third reviewer (M.A.) was consulted.

### 2.5. Methodological Quality Appraisal

The methodological quality of the included studies was assessed in accordance with the methodological principles of systematic reviews [33,34]. The assessment was conducted using the JBI checklists, applying specific tools according to the study design of each included work [34]. In line with the recommendations of the JBI tools, the methodological quality was not summarised using aggregated scores. Instead, the appraisal was interpreted descriptively, highlighting key methodological strengths and potential sources of bias across studies, consistent with approaches adopted in other mixed-methods systematic reviews [37,38]. For mixed-methods study designs, methodological quality was assessed using the Mixed Methods Appraisal Tool (MMAT, 2018 version) [39]. In accordance with the tool’s recommendations, no overall score was calculated; each criterion was rated as “yes,” “no,” or “unclear,” and the results were reported descriptively. The analysis considered the qualitative and quantitative components separately, as well as the integration of findings, to provide a structured and transparent assessment of the risk of bias across methodological domains. Two independent reviewers (L.C., M.D.F.) conducted the assessment process; any discrepancies were resolved by consensus to ensure adequate inter-rater reliability. No studies were excluded from the review based on their methodological quality assessment scores; the appraisal was used to support the interpretation of findings rather than as an exclusion criterion.

### 2.6. Data Extraction

Data were extracted from the included studies using a standardised form developed by the authors. Extracted variables included: author and year of publication, study design, setting and population characteristics, sample size, type of POCUS application, and main clinical, procedural, diagnostic, and professional outcomes. Additional information on training pathways, level of operator experience, and reported limitations was also collected when available, to provide a comprehensive understanding of both effectiveness and implementation. The selection of these variables was guided by the objectives of the review and aimed to capture the multidimensional impact of POCUS, including its role in clinical decision-making, technical performance, diagnostic accuracy, and professional and organisational aspects.

### 2.7. Data Synthesis

Given the heterogeneity of the included studies in terms of design, populations, sample size, and methodologies, a meta-analysis was not considered appropriate. Therefore, a narrative synthesis was conducted. A convergent integrated approach, in line with the JBI guidance for mixed methods systematic reviews, was adopted, whereby quantitative and qualitative findings were initially analysed separately and subsequently integrated to provide a structured and comprehensive interpretation of the evidence. Quantitative findings were transformed into narrative summaries to enable comparison and integration with qualitative data, in line with JBI guidance for convergent integrated mixed-methods reviews [36]. Subsequently, both types of evidence were analysed through an inductive thematic synthesis process. The development of the thematic domains followed an inductive and iterative analytical approach: extracted data were first coded according to their content and meaning, then grouped into conceptually similar categories, and progressively refined through comparison across studies to generate higher-order domains. The coding and categorisation process was conducted by two reviewers and refined through discussion to ensure consistency and reliability of the analysis. No formal weighting of studies was applied; instead, the synthesis considered methodological characteristics, consistency of findings, and the relevance of each study within the identified domains.

## 3. Results

### 3.1. Search Results

A total of 1613 records were identified through database searching, including 429 from PubMed, 362 from Scopus, 597 from CINAHL, and 225 from Web of Science. Of these, 51 articles were considered eligible for full-text assessment. Among these, 9 studies were excluded due to lack of relevance to POCUS, 22 due to an inappropriate study design, 7 because they referred to a pediatric or neonatal population, and 2 due to the unavailability of the full text. The selection process resulted in the final inclusion of 11 studies.

**Figure 1:**
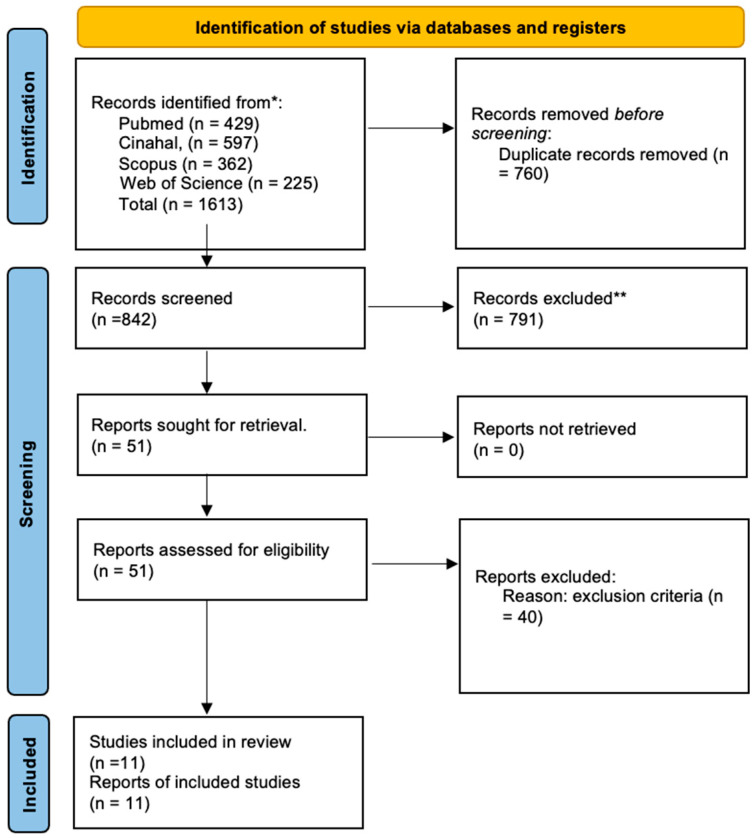
PRISMA flow diagram of the review process. * Consider, if feasible to do so, reporting the number of records identified from each database or register searched (rather than the total number across all databases/registers). ** If automation tools were used, indicate how many records were excluded by a human and how many were excluded by automation tools.

The entire process of identification, screening, eligibility assessment, and study inclusion was documented and graphically represented using the PRISMA 2020 flow diagram (Figure 1).

### 3.2. Study Characteristics

The literature search resulted in the inclusion of eleven studies, published between 2018 and 2025, that evaluated the use of nurse-performed PoCUS in intensive care settings. The limited number of included studies, together with their predominantly observational and single-centre design, should be considered when interpreting the findings. Conducted across multiple geographical regions, including Europe [25,40,41,42], North America [43,44,45,46], South America [47], and Asia [48], these studies reflect a growing international interest in the integration of PoCUS into advanced nursing practice within critical care environments.

In line with this emerging field of research, the included studies present diverse methodological designs and comprise one randomized controlled trial [40], four prospective observational or cohort studies [41,43,46,49], a descriptive prospective correlational study focused on diagnostic accuracy [44], a quality improvement initiative [45], a case series [50], one exploratory mixed-methods study [47] and two qualitative exploratory studies [25,48]. Such variability reflects the still-evolving stage of the implementation of nurse-performed PoCUS, with research focused both on assessing clinical effectiveness and on understanding the processes involved in introducing the technology into care practice.

Across the included studies evaluating critically ill populations, a total of 369 adult ICU patients were represented. Specifically, 114 patients were included in the randomized controlled trial by Bridey et al. (2018) [40], 65 patients in the prospective observational study by Smits et al. (2023) [41], 15 patients in the case series by Corcoran et al. (2023) [50], and 175 patients in the prospective cohort study by Leon et al. (2025) [49]. These studies were conducted in tertiary and academic intensive care settings and involved adult critically ill patients undergoing ultrasound-guided procedures or bedside ultrasound assessments as part of routine or protocolized clinical management.

In addition to patient populations, several studies reported the characteristics of healthcare professionals performing ultrasound examinations or ultrasound-guided procedures. Overall, at least 53 critical care nurses were involved across the studies that explicitly reported operator characteristics. This included 21 nurses who completed ultrasound training in the study by Schott et al. (2024) [46], 5 ICU nurses participating in the quality improvement initiative described by Hartley et al. (2024) [45], 9 critical care nurses involved in the qualitative investigation by Hansen and Solbakken (2024) [25], and 18 ICU nurses included in the qualitative study conducted by Su et al. (2025) [48]. These findings underline the growing role of trained nurses in the integration of point-of-care ultrasound within critical care practice.

Across the included studies, a total of 277 ultrasound-related procedures were reported. These procedures comprised 102 thoracic ultrasound examinations, 15 cardiopulmonary ultrasound scans, 76 ultrasound-guided peripheral intravenous catheter placements, and 84 ultrasound-guided vascular access attempts. Overall, the included studies demonstrated a wide range of clinical applications of nurse-performed point-of-care ultrasound, encompassing both diagnostic bedside assessment and procedural guidance in the management of critically ill patients. To ensure a systematic and coherent synthesis of the included studies, data were extracted and organised in Table 1.

Additional contextual and patient-level characteristics, including severity of illness, use of mechanical ventilation, and clinical conditions, are reported in Table S2 of the Supplementary Materials, where available, to improve the interpretability of the findings across studies.

### 3.3. Quality of Included Studies

The methodological quality of the included studies was assessed using the JBI critical appraisal tools and the MMAT, according to study design.

Overall, the included studies demonstrated variable methodological characteristics across different domains. Randomised and diagnostic accuracy studies generally showed more structured designs, particularly in terms of clearly defined outcomes and systematic data collection processes, although limitations related to sample size and external validity were still present.

Observational and cohort studies displayed greater variability. While some studies adequately addressed key domains such as outcome measurement and data collection, others were limited by potential selection bias, lack of control for confounding factors, and limited use of blinding procedures.

Quality improvement initiatives and case series were inherently limited by their design, particularly due to the absence of control groups and reduced generalisability, although they provided valuable insights into feasibility and real-world implementation.

Qualitative and mixed-methods studies generally demonstrated methodological coherence in terms of study design, data collection, and analytical approach. However, their findings were context-dependent and based on relatively small samples, which may limit transferability. Across studies, the most common sources of potential bias included small sample sizes, single-centre designs, selection bias in non-randomised studies, incomplete adjustment for confounders, and limited use of blinding.

No studies were excluded based on methodological quality. Instead, the appraisal findings were used to inform a cautious interpretation of the results, particularly in relation to the overall strength and certainty of the evidence.

A detailed domain-specific summary of the methodological assessment of the included studies is provided in Supplementary Materials (Table S3).

### 3.4. Synthesis of Results

The results were synthesised using a narrative thematic approach with an inductive process of data comparison and grouping. Extracted outcomes were systematically examined across studies and coded according to their content, and subsequently organised into conceptually similar categories. The coding and categorisation process was conducted by two reviewers and refined through discussion to ensure consistency and reliability of the analysis. Through this iterative process, broader themes were identified and refined to reflect the main areas of impact of nurse-performed POCUS in intensive care. This approach enabled the organisation of heterogeneous evidence into four primary domains: (1) support for the clinical decision-making process, (2) technical performance and procedural outcomes, (3) diagnostic accuracy, and (4) professional autonomy, training, and sustainability of competencies. These domains provide a structured descriptive synthesis of the available evidence and do not imply uniform strength of evidence across all areas. Taken together, these findings should be interpreted with caution, as the available evidence does not permit definitive conclusions and is considered preliminary. Overall, the certainty of evidence across the four domains is low to moderate, given the predominance of observational designs, small sample sizes, and heterogeneity in outcomes.

### 3.5. Support for the Clinical Decision-Making Process

The included studies suggest that nurse-performed PoCUS may contribute to the clinical decision-making process in intensive care, particularly in the respiratory and hemodynamic domains.

In the prospective observational study by Smits et al. (2023) [41], conducted in 65 ICU patients who underwent a total of 102 thoracic ultrasound examinations, nurse-performed lung ultrasound suggested a change in clinical management in 27 examinations (26%, 95% confidence interval (CI) 18–36). Almost all these suggested changes (26/27; 96%) were implemented within the following eight hours. Most of the management modifications occurred within the nursing scope of practice (24/43 interventions; 56%, 95% CI 41–71). Fluid balance management was adjusted in 19 cases (44%), including both positive and negative fluid balance optimisation, while a smaller number of interventions involved changes in ventilatory settings or vasoactive therapy titration. In addition, thoracic ultrasound identified pathological findings in 97% of examinations, and in 7% of cases, the initial diagnosis was revised, sometimes leading to urgent therapeutic interventions. These findings suggest that nurse-performed PoCUS may have a role in informing bedside clinical decision-making and in prompting timely management adjustments in critically ill patients [41].

Convergent results were reported in the case series by Corcoran et al. (2023) [50], which included 15 critically ill patients with COVID-19 who underwent nurse-performed cardiac and lung ultrasound examinations. In this study, ultrasound findings were associated with changes in clinical management in 10 of the 15 cases (67%). The therapeutic adjustments included targeted fluid removal strategies, modifications in respiratory management, and referrals for further cardiological evaluation. In the remaining five cases (33%), the ultrasound examination confirmed the appropriateness of the therapeutic strategy already in place, thereby appearing to support the ongoing clinical decision-making process [50].

In addition to the direct effects on therapeutic decision-making, the qualitative study by Su et al. (2025) [48], which included 18 intensive care nurses, reported that the use of PoCUS was perceived to improve nurses’ ability to identify early signs of clinical deterioration and to participate more actively in interdisciplinary decision-making processes. Participants described PoCUS as a tool that facilitated more effective communication with physicians and supported more timely clinical interventions. Furthermore, nurses reported that ultrasound contributed to making clinical assessment more objective and immediately actionable in nursing care, strengthening their confidence in bedside evaluation and patient monitoring [48].

Overall, the available studies suggest that PoCUS may function as more than a descriptive tool and may be integrated into bedside decision-making processes; however, this interpretation should be viewed cautiously, given the observational and qualitative nature of much of the evidence [41,48,50].

### 3.6. Technical Performance and Procedural Outcomes

On the procedural side, the available studies suggest a variable impact of nurse-performed PoCUS depending on the type of procedure and the organisational context. In peripheral venous access, studies that have implemented structured training programs report improvements in selected technical performance outcomes. In the study by Schott et al. (2024) [46], the success rate of ultrasound-guided peripheral intravenous insertion was 77.4% compared with 35.9% using the traditional landmark technique (*p* < 0.001). The ultrasound-guided approach was also associated with a lower mean number of attempts (1.2 vs. 1.5 attempts; *p* < 0.01). Across 148 insertion attempts in 108 patient encounters, ultrasound guidance rescued 27 failed landmark attempts, while only one case showed the opposite pattern. This corresponds to an absolute success difference of more than 40%, suggesting a potentially clinically relevant advantage in patients with difficult venous access [46].

Similarly, in the quality improvement initiative described by Hartley et al. (2024) [45], a structured ultrasound-guided peripheral intravenous catheter (USG-PIVC) training program was implemented among ICU nurses. The initiative initially involved 5 nurses performing 76 ultrasound-guided insertions, and the program was subsequently expanded to 13 trained nurses (approximately 17% of ICU staff). Success rates reached 70–90% within two attempts, suggesting that the technique may be feasible and potentially effective when implemented within a structured educational and organisational model [45].

However, in the randomised controlled trial by Bridey et al. (2018) [40] conducted on 114 ICU patients with anticipated difficult venous access, no significant differences emerged between the ultrasound-guided and landmark techniques in the median number of insertion attempts (2 vs. 2; *p* = 0.911) or in catheter duration (median 3 days in both groups; *p* = 0.719). Similarly, the first-day success rate was 66% in the ultrasound group and 70% in the landmark group (*p* = 0.84), and the overall success rate was comparable (98% vs. 95%; *p* = 0.618). Although a higher rate of extravasation was observed in the ultrasound group (34% vs. 18%), this difference did not reach statistical significance (*p* = 0.094). These findings suggest that any potential clinical benefit of POCUS-guided peripheral venous access may be influenced by patient complexity and by the level of operator training and experience [40].

In arterial cannulation, the prospective study by León et al. (2025) [49] involving 175 critically ill patients (89 palpation vs. 86 ultrasound-guided procedures) did not show statistically significant differences between the ultrasound-guided technique and palpation in terms of first-attempt success (58% vs. 50.6%; *p* = 0.39; 95% CI −23.4% to +8.3%), total number of attempts (median 1 in both groups), procedure time (350 s vs. 284 s; *p* = 0.44), or complications. The failed attempt rate was lower in the ultrasound group (14% vs. 21.3%; *p* = 0.28), although this difference did not reach statistical significance. Catheter durability was also comparable between groups (approximately 8 days). In this context, ultrasound guidance appeared broadly comparable to traditional practice, with no clear evidence of superiority [49].

Overall, the available evidence suggests that the contribution of nurse-performed PoCUS to procedural outcomes may be more apparent in peripheral venous access procedures, particularly in patients with difficult access and when the method is integrated into structured training programs, while it appears less marked in arterial procedures, where the ultrasound-guided technique shows results comparable to traditional practice [40,45,46,49].

### 3.7. Diagnostic Accuracy

Across the included studies, no clear increase in complications was reported in association with nurse-performed PoCUS in the procedures and bedside assessments examined. In the diagnostic setting, the accuracy of nurse-performed ultrasound assessment appears generally acceptable when supported by structured training pathways, although it does show some variability related to the patient’s clinical condition and the operator’s experience.

The study by Schallom et al. (2020) [44] evaluated the use of ultrasound for bladder volume assessment in critically ill patients. The analysis included 73 ICU patients across four intensive care units, many of whom presented complex clinical conditions (77% receiving dialysis and 28% with ascites). The authors reported that the accuracy of ultrasound-based bladder volume measurements may be influenced by patient-related factors such as ascites, obesity, and complex abdominal conditions, as well as by the operator’s level of technical expertise. Overall, ultrasound measurements showed clinically acceptable agreement with intermittent catheterisation, with the lowest bias observed using the oblique sagittal measurement (−1.3 mL). When applying a clinical decision threshold of ≥300 mL, bladder scanners correctly identified the need for catheterisation in 94–100% of cases, while ultrasound showed greater accuracy at lower volume thresholds (<150 mL), reaching 97–100% accuracy, particularly in patients with ascites. Despite these limitations, ultrasound was described as a non-invasive and repeatable bedside method that can support clinical decision-making and may contribute to reducing unnecessary invasive procedures in the evaluation of urinary retention or bladder distension [44]. Additional supporting data come from technical accuracy studies, which show that adequately trained nursing staff can acquire and interpret ultrasound images with high levels of reliability. Brunhoeber et al. (2018) [43] evaluated the feasibility of nurse practitioner–performed ultrasound assessment of the inferior vena cava (IVC) for estimating volume status in critically ill patients. The study involved 8 acute care nurse practitioners performing 50 IVC ultrasound examinations in a surgical ICU. Image acquisition accuracy reached 86% (93.1% when full physician agreement was considered), while the accuracy of volume status interpretation based on IVC assessment was 80.6% (73.9% with full physician agreement). These findings suggest that, when performed by appropriately trained nurses, bedside ultrasound may achieve a clinically acceptable level of diagnostic reliability for the assessment of intravascular volume status in intensive care settings [43].

Overall, the available studies suggest that the safety and diagnostic reliability of nurse-performed PoCUS may be closely linked to the operator’s level of competence and to the availability of shared protocols and structured training pathways. The adoption of standardised training programs and clinical supervision systems therefore, appears likely to be important to ensuring the safe and effective use of this tool in everyday clinical practice. [43,44]. Across the identified domains, findings were generally consistent in suggesting a potential role of nurse-performed PoCUS; however, variability in study design, sample size, training pathways, and clinical context limits direct comparability across studies and the strength of cross-study inferences.

### 3.8. Professional Autonomy, Training, and Sustainability of Skills

Four of the included studies suggest a potentially important professional impact associated with the implementation of nurse-performed PoCUS. The qualitative studies by Hansen and Solbakken (2024) and by Su et al. (2025) suggest that the use of PoCUS may contribute to increased clinical confidence, situational awareness, and participation in interdisciplinary decision-making processes. The method is frequently described as a tool that makes clinical assessment more objective, facilitates dialogue with the medical team, and may strengthen the perception of professional autonomy and advanced competence within the care team [25,48]. At the same time, however, critical elements are emerging that may limit the sustainability of implementation. Among the main reported barriers are the limited availability of ultrasound equipment, the need for continuous clinical supervision in the initial phases of use, the difficulty of maintaining skills over time, and the absence, in many settings, of formally recognised training and certification pathways. These factors may contribute to discontinuous use of the method and may reduce the effectiveness of implementation programs if they are not adequately supported by institutional policies and dedicated organisational models [25]. The introduction of nurse-performed PoCUS in intensive care does not produce only measurable technical effects but is part of a broader process of organisational and professional evolution. The effectiveness and sustainability of this transformation appear likely to depend on the integration of clinical skills, structured training programs, and appropriate models of institutional governance capable of supporting the stable adoption of the method in everyday care practice [25,45,46,48].

Training appears to be a central element for the effective and safe implementation of nurse-performed PoCUS in the intensive care setting. The included studies show that improvements in procedural performance and diagnostic reliability are closely linked to the presence of structured training pathways and clinical supervision. In the study by Schott et al. (2024), an asynchronous training model integrated with supervised practice led to a significant increase in the success rate of ultrasound-guided peripheral venous access (77.4% vs. 35.9%; *p* < 0.001), with 81% of training activities completed outside working hours, suggesting good organisational sustainability [46]. Similarly, Hartley et al. (2024) report that programs including teaching, simulation, and supervised practice make it possible to achieve success rates between 70% and 90% within two attempts, reducing variability between operators [45]. Qualitative studies also show that decisional security and confidence in using the method depend on the continuity of practical training and the availability of clinical supervision, while the lack of formalised certification pathways may promote a non-uniform adoption of the technique. The accuracy of ultrasound assessment, as demonstrated in the study on bladder volume, is also influenced by the operator’s experience [25,48]. Overall, the available evidence suggests that training is an important prerequisite to ensure the effectiveness, safety, and sustainability of integrating POCUS into intensive nursing practice.

## 4. Discussion

This systematic review aimed to critically synthesise the available results on the use of PoCUS in intensive care units for critically ill adults, evaluating its integration into clinical and nursing practice and its impact on technical, decision-making, organisational, and professional outcomes. Overall, the findings suggest that nurse-performed PoCUS may be integrated into intensive care nursing practice. However, this interpretation should be considered in light of the available evidence, which is predominantly based on observational and single-centre studies. In a clinical setting characterised by hemodynamic instability and the need for timely decisions, the availability of bedside ultrasound data may contribute to supporting clinical assessment and decision-making processes [49,50,51,52]. In this context, nurse-performed PoCUS may represent an additional tool to support nursing clinical reasoning and participation in multidisciplinary care [52]. The availability of bedside ultrasound data may reduce the interval between clinical assessment and care intervention, potentially supporting a timelier decision-making process [36]. However, the observed impact mainly concerns intermediate management changes rather than major clinical outcomes, a factor that calls for a cautious and proportionate interpretation of the results. These interpretations should be viewed considering the predominantly observational and qualitative nature of the included studies, many of which were conducted in single-center settings with limited sample sizes.

On the level of diagnostic reliability, the available data suggest overall acceptable levels of accuracy in applications with a narrow target, such as estimating bladder volume or assessing the inferior vena cava, particularly when the examination is supported by structured training programs [43,44]. In particular, in an environment where fluid management represents a delicate balance between hypoperfusion and overload, access to longitudinal bedside ultrasound data can support more timely and contextualised decisions, reducing exclusive reliance on indirect or delayed parameters [50]. Nevertheless, the variability observed in some dynamic assessments confirms that the reliability of nurse-performed PoCUS is closely dependent both on the complexity of the clinical question and on the operator’s level of experience: in a cardiac intensive care unit, nurses with brief training achieved levels of agreement ranging from moderate to almost perfect compared with physicians in identifying pleural and pericardial effusions and in measuring the diameter of the IVC [29]. The almost perfect agreement for pericardial effusion (AC2 0.95) and the substantial agreement for IVC diameter (0.70) indicate that focused applications, with clear protocols and defined diagnostic targets, may be transferable to nursing practice. The currently available data do not report an increase in complications related to the use of the method, suggesting a generally favourable safety profile in the applications considered [51,52].

As regards procedural outcomes, the available evidence suggests that the benefit may be more apparent for ultrasound-guided peripheral venous access in patients with difficult access, an area in which multiple observational studies and quality improvement projects report higher success rates and a reduction in multiple attempts [45,46,49]. However, the only RCT included did not detect statistically significant differences, suggesting that the effectiveness of the method may be substantially influenced by the organisational context and the operators’ level of expertise [40]. Similarly, in arterial cannulation, the ultrasound-guided technique has proven to be substantially comparable to traditional practice, with no results of clear superiority [49]. These elements suggest that any potential impact of nurse-performed PoCUS may depend on the quality of its implementation and the appropriate selection of indications.

On a professional and organisational level, qualitative studies suggest a perceived increase in clinical confidence, situational awareness, and interdisciplinary participation [25,48]. These results are consistent with the quantitative data on decision-making support, suggesting that POCUS may contribute to strengthening the nursing contribution within clinical processes. However, the sustainability of its implementation appears to depend on the availability of structured training pathways, clinical supervision, and dedicated organisational models. In the absence of these elements, the adoption of the method may be uneven. In many settings, ultrasound training remains focused on medical staff, while the development of nursing skills takes place through informal learning [17].

The interpretation of these findings should be considered in light of several methodological limitations of the included studies. In particular, the overall methodological quality was moderate, and most studies were based on single-center observational designs, which may limit the generalizability of the findings. In addition, the absence of major patient-centred outcomes, such as mortality, ICU length of stay, or long-term clinical impact, substantially limits the ability to assess the overall effectiveness of nurse-performed PoCUS beyond intermediate or process-related outcomes. The heterogeneity of ultrasound protocols, training pathways, and study populations also limits direct comparability across studies. The heterogeneity of the included studies further limits the ability to draw strong conclusions regarding the overall certainty of evidence across the identified domains.

Despite these limitations and the uncertainty of the available evidence, these considerations are closely linked to the methodological quality of the included studies, which directly influences the strength and reliability of the conclusions.

The findings suggest that nurse-performed PoCUS may represent a promising approach to extending bedside diagnostic capabilities within a supervised and collaborative clinical setting; however, this interpretation should be considered with caution, given the limited strength and heterogeneity of the available evidence. Therefore, any implications for clinical practice should be interpreted cautiously until more robust evidence becomes available. From a clinical perspective, the findings suggest that nurse-performed PoCUS may support specific aspects of bedside assessment and procedural practice, particularly when implemented within structured training and supervision frameworks; however, its impact on major patient-centred outcomes remains uncertain. From a theoretical perspective, these findings may be interpreted within the broader context of evolving nursing competencies, although this interpretation should be considered cautiously given the current limitations of the evidence base.

Several studies indicate potential benefits in terms of support for clinical decision-making and improvements in specific technical procedures, while also suggesting a possible contribution of PoCUS to the development of advanced nursing competencies in intensive care settings. However, the available data remain limited and should be interpreted carefully. Further multi-center studies and more robust study designs are needed to better clarify the potential clinical impact of nurse-performed PoCUS, particularly regarding patient-centred outcomes. Future research should also aim to better define the scope, safety, and clinical appropriateness of specific nurse-performed ultrasound procedures across different care settings, particularly distinguishing between procedural and diagnostic applications and their associated risk profiles.

Despite its potential benefits, several challenges should be considered. POCUS is highly operator-dependent and may increase the risk of misinterpretation when training or experience is limited. Its integration into nursing practice may also raise issues related to the scope of practice, role boundaries, and interprofessional dynamics, particularly in settings without well-defined regulatory frameworks. Moreover, as a focused diagnostic tool, POCUS does not replace comprehensive imaging and may be insufficient in complex clinical scenarios. Organisational and logistical constraints, including equipment availability, costs, and the need for ongoing training, as well as time pressures within clinical workflows, may further limit its implementation. Finally, overreliance on ultrasound findings without adequate clinical integration represents an additional risk.

### 4.1. Strengths and Limitations

This review has several notable strengths. First, the study was conducted following a systematic and transparent methodological approach, allowing the identification, appraisal, and synthesis of the available literature on nurse-performed PoCUS in intensive care settings. The inclusion of studies with different methodological designs (prospective comparative, observational, qualitative, and mixed methods) enabled a multidimensional analysis of the phenomenon, integrating measurable technical outcomes with experiential, organisational, and professional dimensions. The convergent synthesis approach further allowed the integration of quantitative and qualitative findings, contributing to a more comprehensive understanding of the role of PoCUS in nursing practice. Importantly, the review focused specifically on adult intensive care settings, a context characterised by high clinical complexity, thereby providing findings that are directly relevant for clinical practice. In addition, the analysis of training pathways and learning processes offers practical insights that may support the implementation of nurse-performed PoCUS in critical care environments.

Despite these strengths, several limitations should be considered when interpreting the findings. First, the diversity of the methodological designs of the included studies, together with the predominance of observational studies and the relatively small sample sizes of several studies, may limit the overall robustness of the available data. Second, although multiple major databases were searched, EMBASE was not included in the search strategy, which may have resulted in the omission of relevant studies. Third, the geographical distribution of the included studies was uneven, with most research conducted in high-income countries in Europe and North America, while research from other regions remains limited. This suggests that the current literature may reflect specific organisational and training contexts, potentially influencing the implementation and reported outcomes of nurse-performed PoCUS. Finally, the absence of major patient-centred outcomes, such as mortality, ICU length of stay, or long-term clinical impact, substantially limits the ability to assess the overall effectiveness of nurse-performed PoCUS beyond intermediate or process-related outcomes.

These limitations have important implications for interpreting the findings. The predominance of observational and single-centre studies, together with small sample sizes and heterogeneous outcomes, limits causal inference, reduces generalizability, and hinders comparability across studies. As a result, the conclusions should be interpreted with caution and considered preliminary rather than definitive.

### 4.2. Implications for Clinical Practice

The findings emerging from this review suggest several relevant implications for clinical practice in intensive care. First, the integration of POCUS into nursing assessment can contribute to a timelier and more dynamic evaluation of the critically ill patient, helping to reduce the gap between clinical observation and care intervention. The systematic use of thoracic and vascular ultrasound at the bedside can support more targeted decisions in the ventilatory, hemodynamic, and infusion domains, improving the personalization of care.

In the procedural field, the adoption of ultrasound-guided peripheral venous access should be particularly considered for patients with difficult access, as it is associated with higher first-attempt success and a reduction in multiple attempts. This can translate into improved patient comfort, reduced local trauma, and a more appropriate use of invasive resources.

From an organisational perspective, the results of the included studies highlight the need for structured training programs, with clinical supervision and standardised competencies. The integration of PoCUS into intensive nursing practice requires a clear definition of certification pathways, ongoing professional development, and adequate availability of devices. Flexible training models, including asynchronous learning, can facilitate implementation without compromising the care workflow.

Finally, the adoption of PoCUS can help strengthen nursing decision-making autonomy and interdisciplinary collaboration, promoting a more integrated model of care oriented toward diagnostic proximity. However, its implementation should be accompanied by appropriate training, monitoring of clinical outcomes, and quality assessments, to ensure safety, appropriateness, and long-term sustainability.

Taken together, the available findings suggest that nurse-performed PoCUS can be considered an integral component of an advanced intensive care model, in which diagnostic proximity, timely decision-making, and professional autonomy converge toward a more personalised management of the critically ill adult patient. Nonetheless, the full integration of this technology requires structured investments in advanced training, clinical governance, competency standardisation, and high-quality research, so that the transformative potential observed can be translated into measurable and sustainable clinical benefits over the long term.

## 5. Conclusions

The present review suggests that nurse-performed PoCUS in the intensive care setting represents an intervention with transformative potential, capable of simultaneously influencing technical performance, decision-making processes, and care organisation. The available findings indicate a significant improvement in procedural performance, particularly in ultrasound-guided peripheral venous access, as well as a tangible impact on clinical management through timely changes in care decisions. At the same time, qualitative studies highlight a strengthening of clinical reasoning, situational awareness, and nurses’ professional autonomy. Taken together, these findings suggest that PoCUS does not merely represent an additional technical skill, but rather a tool integrated within the intensive care model, consistent with the evolution of advanced competencies in critical care. However, the methodological heterogeneity of the included studies and the limited availability of long-term clinical outcomes require cautious interpretation of the results. Multi-center-controlled studies, cost-effectiveness analyses, and standardised training programs are needed to further clarify the clinical impact of nurse-performed PoCUS and support its safe and consistent implementation. A structured investment in education and clinical governance appears essential to translate the observed potential into measurable and sustainable clinical benefits for the adult critically ill patient.

## Figures and Tables

**Table 1:** Main characteristics of the included studies.

Author (Year)	Country	Design	Sample	PoCUS Application	Key Finding
Bridey et al. (2018) [40]	France	RCT	114 ICU patients	US-guided PIVC	No significant difference vs. landmark technique; similar success rates
Brunhoeber et al. (2018) [43]	USA	QI study	8 NPs, 50 scans	IVC assessment	Good accuracy in image acquisition (86%) and interpretation (80.6%)
Corcoran et al. (2023) [50]	UK	Case series	15 ICU patients	Cardiac & lung US	Management changed in 67% of cases
Galon et al. (2025) [47]	Brazil	Mixed-methods	10 nurses	Multi-application PoCUS	Improved safety, decision-making, and professional autonomy
Hansen & Solbakken (2024) [25]	Norway/Sweden	Qualitative	9 nurses	US-guided PIVC	Improved procedural confidence and independence
Hartley et al. (2024) [45]	Canada	QI study	5–13 nurses	US-guided PIVC	High success rates (70–90%) after training
León et al. (2025) [49]	Spain	Cohort	175 ICU patients	Arterial catheterisation	Comparable performance vs. palpation technique
Schallom et al. (2020) [44]	USA	Observational	73 ICU patients	Bladder US	Clinically acceptable accuracy vs. catheterization
Schott et al. (2024) [46]	USA	QI study	36 nurses	US-guided PIV	Higher success vs. anatomy-based approach (77.4% vs. 35.9%)
Smits et al. (2023) [41]	Netherlands	Observational	65 ICU patients	Thoracic US	Management changed in 26% of examinations
Su et al. (2025) [48]	China	Qualitative	18 nurses	Multi-application PoCUS	Improved decision-making, confidence, and autonomy

## Data Availability

No new data were created or analyzed in this study. Data sharing is not applicable.

## Supplementary Materials

The following supporting information can be downloaded at: https://www.mdpi.com/article/10.3390/healthcare14101286/s1, Table S1: complete search strategies for all databases; Table S2: data extraction; Table S3: assessment of the quality of the included studies.

## Abbreviations

The following abbreviations are used in this manuscript:

POCUS	Point-of-care ultrasound
ICU	Intensive Care Unit
DIVA	Difficult Intravenous Access
