# Supplementary material for: Impact of Nurse-Performed Point-of-Care Ultrasound (PoCUS) in Adult Intensive Care: A Systematic Review
Source: Healthcare (Basel). 2026 May 9;14(10):1286. doi: 10.3390/healthcare14101286 (PMC13205619; doi:10.3390/healthcare14101286)
Supplement: Supplementary file 1 [file healthcare-14-01286-s001.zip › healthcare-4230957-SI.pdf]

Supplementary Materials

Table S1: Search Strategy for all databases

DATABASE	SEARCH STRING	N. OF ARTICLES
PUBMED	("Intensive Care Units"[Mesh] OR "Critical Care"[Mesh] OR ICU[tiab] OR "intensive care"[tiab] OR "critical care"[tiab]) AND ("Nurses"[Mesh] OR "Nursing"[Mesh] OR nurse*[tiab] OR "critical care nurse*"[tiab] OR "intensive care nurse*"[tiab]) AND ("Ultrasonography"[Mesh] OR "Point-of-Care Systems"[Mesh] OR ultrasound[tiab] OR ultrasonograph*[tiab] OR "point-of-care ultrasound"[tiab] OR POCUS[tiab] OR "bedside ultrasound"[tiab])	429
CINAHL	((MH "Intensive Care Units+") OR (MH "Critical Care+") OR ICU OR "intensive care" OR "critical care")AND((MH "Nurses+") OR (MH "Nursing+") OR nurse* OR "critical care nurse*" OR "intensive care nurse*")AND((MH "Ultrasonography+") OR (MH "Point-of-Care Testing+") OR ultrasound OR ultrasonograph* OR "point-of-care ultrasound" OR POCUS OR "bedside ultrasound")	597
SCOPUS	TITLE-ABS-KEY (("intensive care" OR ICU OR "critical care") AND (nurse* OR "critical care nurse*" OR "intensive care nurse*") AND ("point-of-care ultrasound" OR POCUS OR ultrasound OR ultrasonograph* OR "bedside ultrasound"))	362
WEB OF SCIENCE	TS= (("intensive care" OR ICU OR "critical care") AND (nurse* OR "critical care nurse*" OR "intensive care nurse*") AND ("point-of-care ultrasound" OR POCUS OR ultrasound OR ultrasonograph* OR "bedside ultrasound"))	225

Search	Actions	Details	Query	Results	Time
#40			Search: **("Intensive Care Units"[Mesh] OR "Critical Care"[Mesh] OR ICU[tiab] OR "intensive care"[tiab] OR "critical care"[tiab]) AND ("Nurses"[Mesh] OR "Nursing"[Mesh] OR nurse*[tiab] OR "critical care nurse*"[tiab] OR "intensive care nurse*"[tiab]) AND ("Ultrasonography"[Mesh] OR "Point-of-Care Systems"[Mesh] OR ultrasound[tiab] OR ultrasonograph*[tiab] OR "point-of-care ultrasound"[tiab] OR POCUS[tiab]**	429	17:01:05

			OR **"bedside ultrasound"[tiab])** Sort by: **Most Recent**		
#39			Search: **"bedside ultrasound"[Title/Abstract]** Sort by: **Most Recent**	1,562	16:59:00
#38			Search: **POCUS[Title/Abstract]** Sort by: **Most Recent**	4,099	16:57:47
#37			Search: **"point-of-care ultrasound"[Title/Abstract]** Sort by: **Most Recent**	6,274	16:57:24
#36			Search: **ultrasonograph[Title/Abstract]** Sort by: **Most Recent**	190	16:56:53
#35			Search: **ultrasound[Title/Abstract]** Sort by: **Most Recent**	384,061	16:56:28
#34			Search: **"Point-of-Care Systems"[MeSH Terms]** Sort by: **Most Recent**	23,921	16:56:05
#33			Search: **Ultrasonography[MeSH Terms]** Sort by: **Most Recent**	523,014	16:55:35
#32			Search: **((((((((((((((("Intensive Care Units") OR ("Critical Care")) OR (ICU)) OR ("intensive care")) OR ("critical care")) AND ("Nurses")) OR ("Nursing")) OR (nurse*)) OR ("critical care nurse*")) OR ("intensive care nurse*")) AND ("Ultrasonography")) OR ("Point-of-Care Systems")) OR (ultrasound)) OR (ultrasonograph*)) OR ("point-of-care ultrasound")) OR (POCUS)) OR ("bedside ultrasound")** Sort by: **Most Recent**	2,277,403	12:59:22
#31			Search: **"bedside ultrasound"** Sort by: **Most Recent**	1,583	12:58:47
#30			Search: **POCUS** Sort by: **Most Recent**	4,160	12:58:16
#29			Search: **"point-of-care ultrasound"** Sort by: **Most Recent**	6,318	12:57:57
#28			Search: **ultrasonograph*** Sort by: **Most Recent**	409,938	12:57:34
#27			Search: **ultrasound** Sort by: **Most Recent**	2,257,992	12:57:13
#26			Search: **"Point-of-Care Systems"** Sort by: **Most Recent**	19,699	12:56:47
#25			Search: **"Ultrasonography"** Sort by: **Most Recent**	400,729	12:56:27

#24			Search: **((((((((("Intensive Care Units") OR ("Critical Care")) OR (ICU)) OR ("intensive care")) OR ("critical care")) AND (Nurses[MeSH Terms])) OR (Nursing[MeSH Terms])) OR ("nurse*"[Title/Abstract])) OR ("critical care nurse*"[Title/Abstract])) OR ("intensive care nurse*"[Title/Abstract])** Sort by: **Most Recent**	555,951	12:55:50
#23			Search: **"intensive care nurse*"[Title/Abstract]** Sort by: **Most Recent**	2,050	12:55:19
#22			Search: **"critical care nurse*"[Title/Abstract]** Sort by: **Most Recent**	4,027	12:54:55
#21			Search: **"nurse*"[Title/Abstract]** Sort by: **Most Recent**	372,436	12:54:32
#20			Search: **Nursing[MeSH Terms]** Sort by: **Most Recent**	273,149	12:54:00
#19			Search: **Nurses[MeSH Terms]** Sort by: **Most Recent**	105,791	12:53:42
#18			Search: **((((((((("Intensive Care Units") OR ("Critical Care")) OR (ICU)) OR ("intensive care")) OR ("critical care")) AND ("Nurses")) OR ("Nursing")) OR (nurse*)) OR ("critical care nurse*")) OR ("intensive care nurse*")** Sort by: **Most Recent**	1,214,602	12:53:05
#17			Search: **"intensive care nurse*"** Sort by: **Most Recent**	2,245	12:52:08
#16			Search: **"critical care nurse*"** Sort by: **Most Recent**	7,461	12:51:52
#15			Search: **nurse*** Sort by: **Most Recent**	525,095	12:51:29
#14			Search: **"Nursing"** Sort by: **Most Recent**	1,031,804	12:51:08
#13			Search: **"Nurses"** Sort by: **Most Recent**	298,434	12:50:37
#12			Search: **(((("Intensive Care Units"[MeSH Terms]) OR ("Critical Care"[MeSH Terms])) OR (ICU[Title/Abstract])) OR ("intensive care"[Title/Abstract])) OR ("critical care"[Title/Abstract])** Sort by: **Most Recent**	362,309	12:50:00
#11			Search: **"critical care"[Title/Abstract]** Sort by: **Most Recent**	55,918	12:49:38
#10			Search: **"intensive care"[Title/Abstract]** Sort by: **Most Recent**	239,949	12:48:58
#9			Search: **ICU[Title/Abstract]** Sort by: **Most Recent**	111,026	12:48:22
#8			Search: **"Critical Care"[MeSH Terms]** Sort by: **Most Recent**	71,290	12:47:51
#7			Search: **"Intensive Care Units"[MeSH Terms]** Sort by: **Most Recent**	123,076	12:46:18

#6			Search: **(((("Intensive Care Units") OR ("Critical Care")) OR (ICU)) OR ("intensive care")) OR ("critical care")** Sort by: **Most Recent**	714,523	12:45:36
#5			Search: **"critical care"** Sort by: **Most Recent**	358,470	12:45:13
#4			Search: **"intensive care"** Sort by: **Most Recent**	427,108	12:44:52
#3			Search: **ICU** Sort by: **Most Recent**	219,582	12:44:03
#2			Search: **"Critical Care"** Sort by: **Most Recent**	358,470	12:43:41
#1			Search: **"Intensive Care Units"** Sort by: **Most Recent**	137,004	12:43:16

Table S2: Data extraction

Authors, Year	Aim(s)	Study design	Sample and setting	Type of intervention	Main results	Declared Limitations
**Bridey et al. (2018) [40]**	To compare the effectiveness of ultrasound-guided peripheral intravenous catheter (PIVC) insertion versus the traditional landmark technique in adult ICU patients with anticipated difficult venous access who no longer required a central venous catheter.	Prospective, randomized, controlled, open-label, single-centre study.	114 adult ICU patients (57 ultrasound-guided group; 57 landmark group). Medical ICU of a tertiary teaching hospital (France).	Ultrasound-guided peripheral venous cannulation performed by critical care nurses (70% trained through structured educational program; certification required ≥4 supervised successful insertions)	**Primary outcome:** No significant difference in number of insertion attempts (median 2 in both groups; p=0.911). **First-day success rate:** 66% (ultrasound) vs 70% (landmark); p=0.84. **Overall success rate**: 98% vs 95%; p=0.618. **Catheter lifespan:** No significant difference (median 3 days; p=0.719). **Complications:** Trend toward higher extravasation in ultrasound group (34% vs 18%; p=0.094). **Satisfaction:** No difference in patient satisfaction; higher nurse-reported suitability with ultrasound guidance.	1) Single-centre design limiting external validity 2) Potentially insufficient nurse training (minimum four supervised insertions) 3)Catheter length possibly inadequate for deeper veins in ultrasound group 4)Sample size may have been underpowered to detect modest differences
**Brunhoeber et al. (2018) [43]**	To evaluate ICU nurse practitioners' ability to accurately acquire and interpret inferior vena cava (IVC) POCUS images for intravascular volume assessment	Prospective quality improvement project.	8 acute care nurse practitioners, 50 IVC ultrasound examinations 22-bed surgical ICU, quaternary academic medical center (USA).	Nurse practitioners performed focused bedside ultrasound assessment of the inferior vena cava to estimate intravascular volume status, with image acquisition and interpretation compared against blinded physician reviewers	**Image acquisition accuracy:** 86% (93.1% when full physician agreement). **Interpretation accuracy:** 80.6% (73.9% with full physician agreement). Discordant interpretations excluded from final calculation.	1)Small convenience sample (n=8 NPs). 2) Single-centre design. 3)No assessment of impact on clinical decision-making. 4)No patient-level outcomes measured. 5)Variable prior POCUS training among participants.
**Corcoran et al. (2022) [50]**	To evaluate the clinical utility of nurse-led focused cardiac and lung point-of-care ultrasound (POCUS) in adult ICU patients with	Single-centre prospective case series	15 adult ICU patients with confirmed COVID-19, 15 combined cardiac and lung POCUS examinations, 80% mechanically ventilated, Median APACHE	An ICU nurse accredited in Focused Ultrasound in Intensive Care (FUSIC) performed combined focused cardiac and six-point lung ultrasound	**Abnormal lung findings:** 100% of patients (irregular pleura, B-lines, subpleural consolidation, hepatization). **Cardiac function:** Normal LV function in 87%; normal RV function in 80%.	1)Small sample size (n=15) 2) Single-centre design 3)No control group 4)Scans performed by one nurse only 5)No external blinded image review 6)Limited generalizability

	confirmed COVID-19 and to assess its contribution to clinical decision-making		II: 18, Median SOFA score: 13<br><br>Adult ICU, tertiary referral hospital (King's College Hospital, London, UK).	examinations using a standardized protocol, with findings integrated into multidisciplinary clinical decision-making.	**Lung severity score:** Moderately correlated with APACHE II (r = 0.70; p = .003).<br>**Change in clinical management:** 67% of cases (10/15).<br>**Targeted fluid removal:** 27%.<br>**Change in respiratory management:** 20%.<br>**Formal echocardiography requested:** 20%.<br>**No intervention required:** 33% of cases.<br>**30-day outcomes:** 33% discharged, 33% remained in ICU, 33% died.	
**Galon et al. (2025) [47]**	To describe the usability of bedside ultrasound (POCUS) as perceived by nurses working in emergency, urgent care, and intensive care units.	Exploratory study using a mixed methods approach	10 certified nurses (Mean ICU experience: 6.1 years; mean POCUS certification time: 9.1 months)<br><br>Three adult ICUs and one emergency/urgent care department in a large tertiary university hospital, Paraná, Brazil.	Certified nurses integrated bedside POCUS into routine clinical practice for procedural support and patient assessment, including vascular access, bladder evaluation, cardiovascular and pulmonary assessment, gastrointestinal procedures, and verification of airway devices.	**Procedures supported:** Peripheral and arterial puncture (100%), bladder assessment (100%), cardiovascular and lung assessment, gastrointestinal procedures, intracranial pressure estimation.<br>**Perceived impact:** Enhanced patient safety, reduced adverse events and material waste, improved clinical reasoning and decision-making.<br>**Professional impact:** Increased autonomy, strengthened professional identity, improved confidence.<br>**Facilitators:** Device availability, real-time imaging, institutional access to technology.<br>**Barriers:** Interprofessional resistance, staffing constraints, technical skill dependency.	1)Single-centre study<br>2)Small sample size (n=10)<br>3)Recent national regulation limiting literature comparison<br>4)Findings based on self-reported perceptions
**Hansen & Solbakken, (2024) [45]**	To explore and describe critical care nurses' experiences	Qualitative study using a hermeneutic approach and reflexive	9 critical care nurses (3 female, 6 male), Mean 14.8 years nursing experience, Mean	Critical care nurses used real-time ultrasound guidance to establish	Five overarching themes were identified under the core concept: "POCUS simplifies a	1)Small sample size (n=9)<br>2)Scandinavian context may limit transferability<br>3)Self-reported experiential data

	and perceptions of using POCUS to establish peripheral intravenous access in ICU patients with difficult intravenous access (DIVA).	thematic analysis.	7.1 years ICU experience, Mean 3.3 years experience with US-guided PIVC<br><br>Six adult ICUs across Norway and Sweden.	peripheral intravenous access in ICU patients with difficult intravenous access, integrating POCUS into routine bedside practice.	complicated procedure":<br>• Improved patient engagement through shared screen viewing and procedural transparency.<br>• Enhanced visualization enabling safer vein selection and more appropriate catheter placement.<br>• Increased nurse independence in managing DIVA without reliance on physicians.<br>• Greater action readiness and perceived impact on timely treatment initiation.<br>Expanded professional role, improved job satisfaction, and strengthened technological identity.	4)Potential researcher pre-understanding due to prior POCUS experience
**Hartley et al. (2024) [45]**	To develop and implement a structured ultrasound-guided peripheral intravenous catheter (USG-PIVC) education program for ICU nurses, evaluate handheld ultrasound device selection, and assess insertion success and program feasibility.	Single-centre quality improvement initiative with descriptive analysis.	5 ICU nurses, 76 USG-PIVC insertions performed on adult patients, subsequent expansion to 13 trained nurses (~17% of ICU staff)<br><br>Adult ICU, Royal Victoria Regional Health Centre, Ontario, Canada.	ICU nurses completed a structured multimodal USG-PIVC training program including asynchronous theoretical education, classroom simulation using a gel-based model, supervised clinical insertions, competency validation, and structured evaluation of three handheld ultrasound	**Total insertions:** 76 across three handheld ultrasound devices.<br>**Overall insertion success rate:** 70–90% within two attempts.<br>**Preferred device characteristics:** Integrated screen-probe design, portability, ease of use, rapid start-up, vendor-supported training.<br>**Education program impact:** High nurse satisfaction; reported increased knowledge, confidence, and perceived improvement in patient care.<br>**Program sustainability:** Expansion to additional	1)Small-scale QI initiative (n=5 nurses initially).<br>2)No direct comparison with traditional palpation method.<br>3) No formal DIVA scale utilization.<br>4) Limited objective complication data.<br><br>5) Potential conflict of interest (device manufacturer involvement in training).

				devices prior to implementation.	ICU nurses; transition to standard practice.	
**Leon et al. (2025) [49]**	To evaluate the effectiveness and safety of ultrasound-guided arterial catheterisation performed by ICU nurses with limited prior ultrasound experience compared with the traditional palpation technique.	Prospective observational cohort study with pre–post training phases (single-centre ICU study).	175 critically ill adult patients. Palpation group (PP): n = 89 Ultrasound group (US): n = 86<br>-26.8% receiving vasoactive support<br>-12.6% with local oedema at insertion site<br>13-bed adult medical ICU, tertiary university hospital (Spain).	ICU nurses performed arterial catheterisation using either the standard palpation technique or real-time ultrasound guidance following a structured training program that included didactic sessions, simulation practice, and supervised procedures.	**First-attempt success rate** (primary outcome):50.6% (PP) vs 58% (US); p = 0.39.<br>**Failed attempt rate:** 21.3% (PP) vs 14% (US); p = 0.28.<br>**Procedure time:** 284 s (PP) vs 350 s (US); p = 0.44.<br>**Number of attempts:** No significant difference (median 1 in both groups).<br>**Complications:** No significant differences in haematoma, catheter dysfunction, or infection.<br>**Catheter durability:** Comparable between groups (≈8 days).<br>Ultrasound-guided arterial catheterisation performed by novice ICU nurses after brief training demonstrated performance and safety comparable to the palpation technique.	1)non-randomised design (potential selection bias)<br>2)Single-centre study<br>3)Nurses on ultrasound learning curve<br>4)Catheter-to-vessel ratio not systematically recorded<br>5)Partial recruitment during COVID-19 pandemic<br>6)Number of punctures during failed attempts not documented
**Schallom et al. (2020) [44]**	To evaluate the accuracy of bladder scanner (BS) and ultrasound (US) bladder volume measurements compared with intermittent straight catheterization (ISC) in ICU patients, and to assess clinician-dependent variability in measurements.	Prospective correlational descriptive study with inter-rater reliability testing.	73 adult ICU patients, 77% receiving dialysis, 23% unable to void.<br>Mean age: 56.9 ± 16.1 years. 28% had abdominal fluid (ascites)<br>Four adult ICUs (medical, surgical/trauma, cardiothoracic, neuro ICU) in a 1350-bed quaternary academic medical center (USA).	Bedside RNs and APRNs performed bladder volume assessment using both bladder scanner (BS) and two-dimensional ultrasound (US) following structured training and inter-rater reliability validation, with measurements compared to intermittent straight catheterization (gold standard).	**Overall accuracy:** Both BS and US provided clinically acceptable bladder volume estimates.<br>**Bias:** Lowest bias observed with oblique sagittal US measurement (−1.3 mL).<br>**Ascites effect:** US more accurate than BS when abdominal fluid present.<br>**Clinical decision threshold ≥300 mL:** BS correctly identified need for catheterization in 94–100% of cases; US less accurate at this threshold.<br>**Threshold <150 mL (no catheterization):** US	1) Single-centre study<br>2) Convenience sample of clinicians<br>3) Limited device generalizability (single vendor)<br>4) Addition of oblique measurement mid-study<br>5) Potential variability in gold-standard catheter drainage

					accuracy 97–100%, particularly superior in presence of ascites. APRNs achieved comparable accuracy to physicians after structured training.	
**Schott et al. (2024) [46]**	To assess the feasibility of an asynchronous educational model for training critical care nurses in ultrasound-guided peripheral intravenous (PIV) placement and to compare procedural success between ultrasound-guided and anatomy-based approaches.	Prospective observational quality improvement study.	36 nurses registered, 21 completed asynchronous training, 8 nurses recorded clinical PIV placements, 108 patient encounters (148 total attempts) Medical and surgical adult ICUs within a Veterans Affairs tertiary care hospital (USA).	Critical care nurses completed a self-directed asynchronous training program using a cart-based simulation system, including a recorded didactic module and supervised competency assessment, followed by real-time ultrasound-guided PIV placement in clinical practice.	**Anatomy-based success rate:** 35.9% (23/64 attempts). **Ultrasound-guided success rate:** 77.4% (65/84 attempts); p<0.001. **Mean number of attempts (successful insertions):** Anatomy: 1.5 (SD 1.0); Ultrasound: 1.2 (SD 0.4); p<0.01. **Cross-over benefit:** 27 cases failed by anatomy but successful with ultrasound; 1 inverse case. **Escalation:** Difficult IV team activated in 9 cases; central venous catheter required in 3 cases. **Educational outcomes:** Significant increase in self-reported confidence with ultrasound use post-training.	1) Single-centre pilot QI design 2) Voluntary participation (selection bias) 3)Self-reported procedural data 4) Small number of nurses applying skill clinically (n=8) 5)No blinded outcome verification
**Smits et al. (2023) [41]**	To determine the impact of nurse-performed thoracic ultrasound (UltraNurse model) on clinical management in adult ICU patients, specifically evaluating the frequency of management changes following	Prospective, single-centre observational study	102 thoracic ultrasound examinations, 65 adult ICU patients, 89% mechanically ventilated, Mean SOFA score: 9<br><br>Mixed medical–surgical academic ICU, Amsterdam University Medical Center (The Netherlands).	Certified ICU nurses (“UltraNurses”) performed standardized thoracic ultrasound examinations using a structured protocol including lung assessment (BLUE protocol) and cardiac output estimation via velocity time integral, integrated into	**Change of management:** 26% of examinations (27/102). **Execution of changes:** 96% implemented within 8 hours. **Scope of changes:** 56% within nursing scope of practice. **Fluid management modification:** 44% of examinations. **Pathology detection:** 97% of examinations (most frequent: atelectasis 58%, pleural effusion 58%,	1)Single-centre design 2)Potential selection bias (clinically indicated ultrasounds only) 3)Possible physician influence on management decisions 4)High proportion of COVID-19 patients 5)Observational design without control group

	ultrasound examination.			routine clinical practice.	pulmonary edema 51%).<br>**Formal diagnosis change:** 7% of cases, some requiring urgent intervention.<br>**Frequency of use:** 1 ultrasound per 4 nursing shifts.	
**Su et al. (2025) [48]**	To explore ICU nurses' experiences in implementing POCUS in clinical practice, identify perceived benefits and barriers, and inform strategies for supporting nurse-led POCUS integration.	Descriptive qualitative study using semi-structured interviews and Colaizzi's seven-step thematic analysis.	18 ICU nurses (13 female, 5 male), ≥3 years ICU experience, Certified in critical care ultrasound, ≥2 years experience using POCUS. Four tertiary (Grade III-A) academic hospitals, Hunan Province, China.	Certified ICU nurses integrated bedside POCUS into routine clinical practice for assessment and procedural support (e.g., vascular access, lung assessment, DVT screening, gastric residual volume, bladder scanning), following standardized national training.	Four overarching themes emerged:<br>• Perceived clinical value: Improved assessment accuracy, enhanced nursing decision-making, more targeted care.<br>• Professional impact: Increased confidence, autonomy, strengthened professional identity, career development opportunities.<br>• Training challenges: Technical complexity, difficulty in image interpretation, need for structured and ongoing supervision.<br>• Implementation barriers: Limited equipment, resource constraints, patient/family misunderstanding, and potential risks (misinterpretation, infection control concerns).	1)Single-region study (China), limiting transferability<br>2)Sample size limited to 18 participants<br>3)Cross-sectional qualitative design<br>4)Findings based on self-reported experiences

Table S3: Assessment of the quality of the included studies

Study Analysed with JBI Critical Appraisal Checklist for quasi-experimental studies

Author and Year	Q1	Q2	Q3	Q4	Q5	Q6	Q7	Q8	Q9	JBI Score
Hartley et al. (2024) [45]	+	N	U	U	N	+	+	+	+	5/9 (56%)

Study Analysed with JBI Critical Appraisal Checklist for cohort studies

Author and Year	Q1	Q2	Q3	Q4	Q5	Q6	Q7	Q8	Q9	Q10	Q11	JBI Score
Brunhoeber et al. (2018) [43]	U	N	+	U	N	N	+	N	N	N	+	3/11 (27%)
Leòn et al. (2024) [49]	+	+	+	+	U	+	+	+	+	N	+	9/11 (82%)
Schott et al. (2024) [46]	+	+	U	-	-	+	U	+	-	-	+	5/11 (45%)
Smits et al. (2023) [41]	+	+	+	U	+	+	+	+	U	N	+	8/11 (73%)

Study Analysed with JBI Critical Appraisal Checklist for RCTs

Author and Year	Q1	Q2	Q3	Q4	Q5	Q6	Q7	Q8	Q9	Q10	Q11	Q12	Q13	JBI Score
Bridey et al. (2018) [40]	+	+	+	N	N	+	N	+	+	+	+	+	+	10/13 (77%)

Study Analysed with JBI Critical Appraisal Checklist for Case Series

Author and Year	Q1	Q2	Q3	Q4	Q5	Q6	Q7	Q8	Q10	Q11	JBI Score
Corcoran et al. (2022) [50]	+	+	+	+	+	+	+	+	+	+	10/10 (100%)

Author and Year	Q1	Q2	Q3	Q4	Q5	Q6	Q7	Q8	Q10	Q11	JBI Score
Hansen et al. (2024) [25]	+	+	+	+	+	+	+	+	+	+	10/10 (100%)
Su et al. (2025) [48]	+	+	+	+	+	N	N	+	+	+	8/10 (80%)

Author and Year	Q1	Q2	Q3	Q4	Q5	Q6	Q7	Q8	Q10	Q11	JBI Score

Schallom et al. (2020) [44]	N	+	+	+	+	+	+	+	+	+	9/10 (90%)

Study Analysed with JBI Critical Appraisal Checklist for Qualitative Research

Study Analysed with JBI Critical Appraisal Checklist for diagnostic test accuracy studies

Study Analysed with MMAT for mix methods studies

Author and Year	Q1	Q2	Q3	Q4	Q5	Q6	Q7	Q8	Q9	Q10	Q11	Q12	Q13	Q14	Q15	Q16	Q17	MMAT Score
Galon et al. (2025) [47]	+	+	+	+	+	+	+	+	N	U	+	+	+	+	+	U	+	Moderate

Note: + = Yes, - = No, U = Unclear, N = Not Applicable, JBI = Joanna Briggs Institute, RCT = randomized controlled trial, MMAT = Mixed Methods Appraisal Tool
